# AdenPredictor: accurate prediction of the adenylation domain specificity of nonribosomal peptide biosynthetic gene clusters in microbial genomes

**DOI:** 10.1093/bioinformatics/btad235

**Published:** 2023-06-30

**Authors:** Mihir Mongia, Romel Baral, Abhinav Adduri, Donghui Yan, Yudong Liu, Yuying Bian, Paul Kim, Bahar Behsaz, Hosein Mohimani

**Affiliations:** Computational Biology, School of Computer Science, Carnegie Mellon, Pittsburgh, PA 15213, United States; Computational Biology, School of Computer Science, Carnegie Mellon, Pittsburgh, PA 15213, United States; Computational Biology, School of Computer Science, Carnegie Mellon, Pittsburgh, PA 15213, United States; Computational Biology, School of Computer Science, Carnegie Mellon, Pittsburgh, PA 15213, United States; Computational Biology, School of Computer Science, Carnegie Mellon, Pittsburgh, PA 15213, United States; Computational Biology, School of Computer Science, Carnegie Mellon, Pittsburgh, PA 15213, United States; Computational Biology, School of Computer Science, Carnegie Mellon, Pittsburgh, PA 15213, United States; Institute for Protein Design, University of Washington, Seattle, WA 8195, United States; Molecular Engineering Ph.D. Program, University of Washington, Seattle, WA 98195, United States; Computational Biology, School of Computer Science, Carnegie Mellon, Pittsburgh, PA 15213, United States; Computational Biology, School of Computer Science, Carnegie Mellon, Pittsburgh, PA 15213, United States

## Abstract

**Summary**Microbial natural products represent a major source of bioactive compounds for drug discovery. Among these molecules, nonribosomal peptides (NRPs) represent a diverse class that include antibiotics, immunosuppressants, anticancer agents, toxins, siderophores, pigments, and cytostatics. The discovery of novel NRPs remains a laborious process because many NRPs consist of nonstandard amino acids that are assembled by nonribosomal peptide synthetases (NRPSs). Adenylation domains (A-domains) in NRPSs are responsible for selection and activation of monomers appearing in NRPs. During the past decade, several support vector machine-based algorithms have been developed for predicting the specificity of the monomers present in NRPs. These algorithms utilize physiochemical features of the amino acids present in the A-domains of NRPSs. In this article, we benchmarked the performance of various machine learning algorithms and features for predicting specificities of NRPSs and we showed that the extra trees model paired with one-hot encoding features outperforms the existing approaches. Moreover, we show that unsupervised clustering of 453 560 A-domains reveals many clusters that correspond to potentially novel amino acids. While it is challenging to predict the chemical structure of these amino acids, we developed novel techniques to predict their various properties, including polarity, hydrophobicity, charge, and presence of aromatic rings, carboxyl, and hydroxyl groups.

## 1 Introduction

Nonribosomal peptides (NRPs) are a class of natural products with diverse applications in medicine and agriculture (Miller and Gulick 2016). NRPs are synthesized by nonribosomal peptide synthetase (NRPS), which are modular assembly lines at minimum consisting of an adenylation domains (A-domains), peptidyl carrier domains (PCP-domains), and condensation domains (C-domains) (Martínez-Núñez and López 2016). Usually, each NRPS module is responsible for the recruitment of a single amino acid into the backbone of an NRP, which is specified by the A-domain. The first 3D structure of an A-domain, responsible for incorporating phenylalanine in Gramacidine S, became available in 1997 (Conti *et al.* 1997). Based on this structure, Stachelhaus *et al.* (1999) constructed an NRP code (in contrast to genetic code) for predicting incorporated monomers based on eight amino acids present in the binding pockets of A-domains. Later, Rausch *et al.* (2005) expanded the binding pocket to 34 amino acids, called A-domain signatures and extracted physiochemical properties of the amino acids as features. They then applied a new algorithm using support vector machines to predict the incorporated monomers of A-domains based on their amino acid sequences. Li *et al.* (2009) designed a webserver for prediction of the structure of NRPs and polyketides from microbial genome sequences. Röttig *et al.* (2011) improved these methods by using an expanded set of physiochemical features and semi-supervised clustering. In SANDPUMA, Chevrette *et al.* (2017) improved the prediction accuracy by introducing an ensemble-based algorithm. In this article, we present AdenPedictor, a machine learning toolkit that provides substrate binding predictions and unsupervised clustering for A-domains. By utilizing the extra trees machine learning model (Geurts *et al.* 2006), AdenPredictor improves prediction accuracy over the state of the art by 8% points. Moreover, by applying unsupervised learning methods on a collection A-domains, AdenPredictor identifies A-domains corresponding to previously unreported amino acids.

In the context of substrate binding predictions, our results show that while the existing methods are accurate in case of A-domains that are very similar to domains with known substrates (present in the original training data), their accuracy drops significantly in case of novel A-domains (domains that are distinct from any domain in the training data). In fact, this is a common shortcoming of machine learning methods with string or graph inputs (Wu *et al.* 2018). To alleviate this problem, we applied various machine learning techniques (e.g. logistic regression, decision trees, random forests, probabilistic learning and graph neural networks) across different features. These features include amino acids in the binding pocket, their physiochemical properties, and their 3D properties predicted by RaptorX (Källberg *et al.* 2012) and Alphafold2 (Jumper *et al.* 2021). Our results show that tree-based machine learning models outperform the existing approaches (Röttig *et al.* 2011, Chevrette *et al.* 2017) in overall accuracy by 8%. In case of A-domains that are significantly different from training data, tree-based methods improve state of the art methods by ∼30%. Our results show that in contrast to the previous reports, using physiochemical features does not improve the performance of machine learning algorithms in comparison to more basic amino acid features.

We further conducted an exhaustive analysis of publicly available A-domains. We extracted 453 560 A-domains from 689 227 microbial genomes available at National Center for Biotechnology Information GenBank repository and clustered them using unsupervised learning techniques. Information visualization of the results shows that many classes of A-domains have unknown substrate specificity: 19 out of the 50 largest clusters do not contain any known A-domains. We hypothesize that these domains are likely to represent novel amino acids with novel chemistry and bioactivities, making them potential leads for drug discovery.

## 2 Data and methods

### 2.1 Datasets

Supervised learning methods are trained on a dataset consisting of 658 sequences of 34 amino acids and corresponding substrate labels. This dataset is generated by first taking 1546 labeled A-domains reported by Chevrette *et al.* (2017) and then aligning them to NRPS A-domain AMP-binding (PFAM ID PF00501.21) with HMMER3 (Eddy *et al*. 2009) (a profile hidden Markov model). The average length of A-domains is 401 amino acids. Then as prescribed by Rausch *et al.* (2005), for each A-domain 34 residues thought to be part of the A-domain binding pocket are selected (see Supplementary Section S5 for further details) and then concatenated together to make the A-domain’s signature. The resulting 1546 signatures are not all distinct, and thus are deduplicated to 658 datapoints. Supplementary Fig. S3 shows the frequency of various signatures. Supplementary Section S6 details number of datapoints belonging to each label.

Unsupervised clustering is conducted on 453 560 A-domains extracted by running antiSMASH (Blin *et al.* 2019) on 689 227 microbial genomes from the National Center for Biotechnology Information GenBank repository. As in the case of A-domains used for supervised learning, each domain is mapped to a length-34 signature.

### 2.2 Encoding schemes

A-domain signatures are mapped to various feature vectors as preprocessing step before supervised and unsupervised learning. Amino acids in the signatures are mapped to either physiochemical features (Rausch *et al.* 2005, Röttig *et al.* 2011), one-hot encoding (OHE) features, RaptorX structural (Källberg *et al.* 2012), RaptorX property features (Wang *et al.* 2016a), or Alphafold2 structural features (Jumper *et al.* 2021). The final feature vector of a A-domain is a concatenation of the mappings of each amino acid present in the signature.

Physiochemical features (used in NPRSPredictor2) consist of 12 AA index (Kawashima *et al.* 2007) descriptors and 3 z-scale descriptors (Wold *et al.* 1987) that represent hydrophobicity, size, and electronic properties. The 12 AA index features are chosen as prescribed by Rausch *et al.* (2005) (see Supplementary Table S1 for further details). OHE features are binary vectors of length 20 where a single entry is zero or one. Each amino acid maps to a unique binary vector. RaptorX and Alphafold2 are deep learning systems that given a protein/A-domain sequence, will predict properties of each amino acid in the sequence. RaptorX and Alphafold2 structural features are locations of each amino acid in 3D space and RaptorX property features include 15 structural and chemical properties of amino acids, including secondary structure type, disordered state, and solvent accessibility predicted by a DeepCNF neural network (Wang *et al.* 2016b).

Unsupervised clustering is conducted using RaptorX property features and supervised learning is conducted with all encoding schemes mentioned above.

### 2.3 Machine learning classifiers

We applied several machine learning classifiers including logistic regression, support vector machine (SVM), k-nearest neighbor, multilayer perceptron, random forest, decision tree, Bernoulli Naive Bayes, Gaussian Naive Bayes, extremely randomized trees, and graph neural networks (You *et al.* 2020) and compared the accuracy of these models to the accuracy of NRPSPredictor2 (Röttig *et al.* 2011), the most widely used and cited tool for A-domain substrate prediction. In order to get consistent estimates of the test set accuracy, we shuffled the data and applied the machine learning classifiers 20 times. In each shuffle, the data are split randomly into training and test sets in 80:20 ratio. We averaged the test accuracy over 20 shuffles. Details of machine learning model parameters are described in the Supplementary Section S1.

### 2.4 Machine learning metrics and generalization

In many learning tasks, it is common to see that the prediction accuracy drops for test data that are more distinct from training data (Wu *et al.* 2018). To evaluate the generalizability of machine learning classifiers, we split the test data points into buckets. For every given positive number *k*, we define $B_{k}$ to be the bucket containing test data points with minimum Hamming distance *k* from any training data point. Thus, buckets with higher *k* represent the test sets containing data points that are more distinct from the training set. Moreover, $B_{k+}$ is defined as the portion of the test data for which the minimum Hamming distance to any training data point is at least *k*. Various methods are benchmarked on different buckets.

### 2.5 Removing bias with weight balancing

In order to remove bias induced by the imbalanced dataset, we apply weight balancing. For each machine learning model, the loss function has the form where *t* is the index of each training point, $y^{t}$ represents the true label of each training point, $x^{t}$ represents the features of each training point, *f* is the classification function, and *L* refers to a loss function that is low when $f(x^{t})$ is similar to $y^{t}$ and high otherwise. In weight balancing, the loss function is modified to be where $b^{t}$ is the number of training points with label $y^{t}$. This way we can avoid bias toward frequent residues. Each label contributes the same amount to the loss function that we aim to minimize.


(1) $$\min\sum_{t=1}^{T}L(f(x^{t}),y^{t}),$$

(2) $$\min\sum_{t=1}^{T}\frac{L(f(x^{t}),y^{t})}{b^{t}},$$

### 2.6 Unsupervised clustering and visualization of unlabeled data

Unlabeled A-domains are mapped to feature vectors consisting of RaptorX property features of amino acids present in each A-domain’s signature (see Sections 2.1 and 2.2 entitled Datasets and Encoding Schemes respectively for further details). The resulting feature vectors are clustered using K-means clustering where the parameter K=200 [decided via the elbow method (Bholowalia and Kumar 2014)] and distance metric is euclidean. Clusters are visualized using t-distributed stochastic neighbor embedding (t-SNE) (van der Maaten and Hinton 2008).

### 2.7 Property prediction of novel amino acids

Classification algorithms that represent the substrate prediction as a output vector with each position in the vector corresponding to the probability that a given amino acid is the substrate for the A-domain, will be unable to make predictions for novel substrates. In order to account for these novel substrates, we have also explored various learning techniques to predict chemical properties of the final amino acid monomer, including polarity, hydrophobicity, charge, and the presence of aromatic ring, carboxyl, or hydroxyl groups. Such methods would allow for researchers to narrow down the identity of the amino acid.

## 3 Results

### 3.1 Benchmarking accuracy and generalization ability of different learning techniques

**Figure 1.**
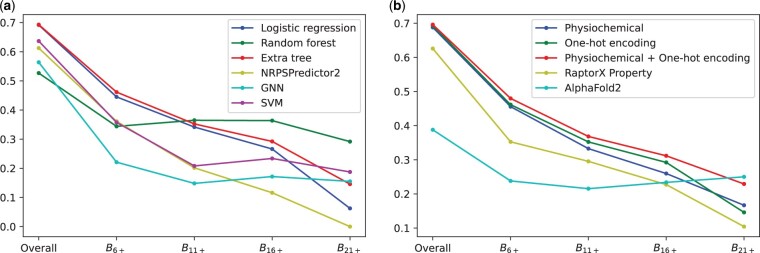
(a) The accuracy of logistic regression, Random forest, Extra tree, NRPSpredictor2 (Röttig *et al*. 2011), and graph neural network (You *et al*. 2020) classifiers using one-hot encoding scheme. Extra tree is 8% more accurate than NRPSPredictor2 on the whole test dataset. (b) The accuracy of extra tree classifier using different encoding schemes.

Figure 1 shows a comparison of accuracy of various machine learning models using OHE features. In order to evaluate the generalization ability of these models, we additionally show each model’s accuracy for various test datasets differing in the degree of dissimilarity with the training data. For every given positive number *k*, we define $B_{k}$ to be the bucket containing test data points with minimum Hamming distance *k* from any training data point. Moreover, $B_{k+}$ is defined as the portion of the test data for which the minimum Hamming distance to any training data point is at least *k*. Buckets with higher *k* represent test sets containing data points that are more distinct from the training set. Various methods are benchmarked on different buckets (Fig. 1a).

Our results show that the extra tree method achieves 69% overall accuracy, in comparison to 61% overall accuracy for NRPSpredictor2—the state of the art model for A-domain substrate prediction. In case of *B*${}_{21+}$ bucket (novel A-domains with less similarity to known A-domains), extra tree achieves 15% accuracy in comparison to 0% for NRPSpredictor2 (Röttig *et al.* 2011). Supplementary Fig. S1 shows the comparison of various methods based on physiochemical and RaptorX features. Supplementary Table S2 shows the fraction of test data belonging to different buckets.

### 3.2 Benchmarking different encoding schemes

Figure 1 shows a comparison of accuracy of extra tree method using OHE, physiochemical (used for NRPSPredictor2), and RaptorX features. Our results show that, in contrast to previous reports (Röttig *et al.* 2011), physiochemical features alone do not provide advantages in accuracy. Extra tree models with OHE features are competitive or better than extra tree models with physiochemical features. Supplementary Fig. S2 shows a comparison of various encoding techniques with extra tree classifier.

### 3.3 Removing bias

**Figure 2.**
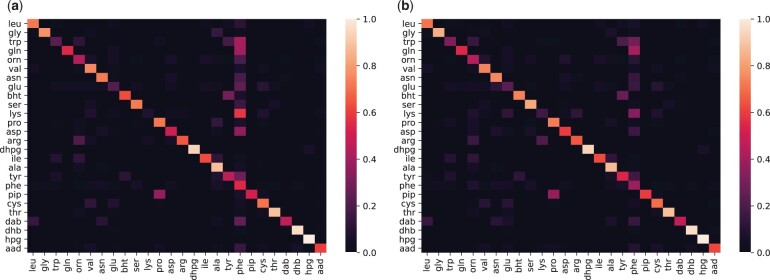
Confusion matrix obtained by using logistic regression paired with one-hot encoding features (a) before and (b) after applying weight balancing.

Currently, a significant portion (10.9%) of training data is from phenylalanine residues. This results in a bias toward predicting phenylalanine (Fig. 2 and supplementary Fig. S5a, c, e, and g). To alleviate this issue, we applied weight balancing. Weight balancing improves the accuracy of prediction for some classifiers. Table 1 shows the change in test accuracy using physiochemical encoding style or OHE style when weight balancing is applied to four classifiers. Figure 2 and Supplementary Fig. S5b, d, h, and f show confusion matrix after application of weight balancing.

**Table 1.** Accuracy of different classifiers using physiochemical (Röttig *et al.* 2011) and one-hot encoding (OHE) before and after applying weight balancing.

Classifier	Before weight balancing		After weight balancing	
	Physiochemical	OHE	Physiochemical	OHE
Logistic regression	0.588	0.692	0.594	0.685
Random forest	0.612	0.527	0.667	0.634
Decision tree	0.615	0.614	0.608	0.603
**Extra tree** ^a^	**0.688**	**0.693**	**0.691**	**0.699**
SVM	0.609	0.637	0.611	0.637

a Method with the highest accuracy (Extra tree) is shown in bold.

### 3.4 Unsupervised clustering of A-domains from microbial genomes

**Figure 3.**
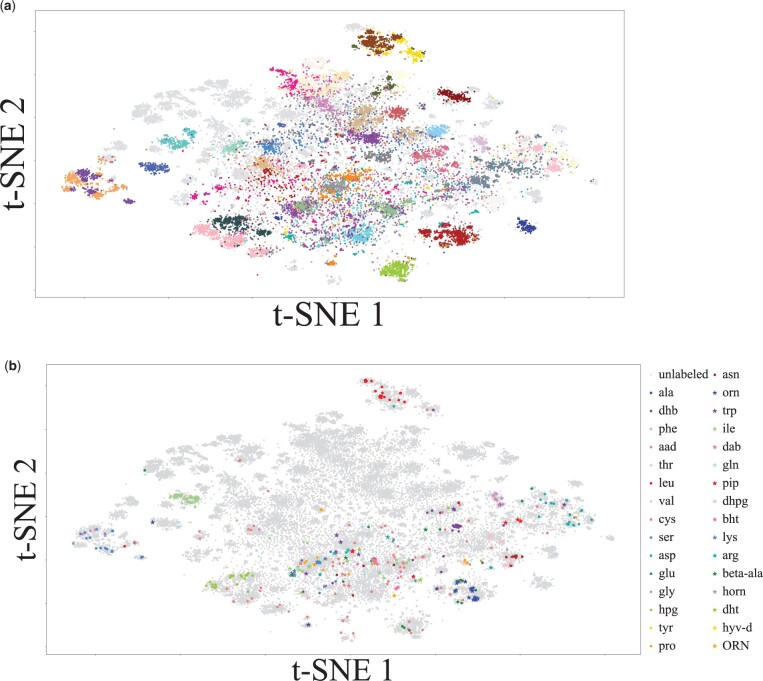
(a) t-SNE visualization of 50 largest clusters constructed by applying k-means on RaptorX encoding of A-domains. All A-domains belonging to a specific cluster are given the same color. (b) t-SNE visualization of all labeled (in color) and unlabeled (gray) A-domains. Labeled A-domains are color-coded by substrates. Several colored regions in (a) are completely grey in (b). Thus the clusters represented by these colored regions do not have any A-domains for which substrate specificity is known and likely represent novel amino acids.

A total of 453 560 A-domains were identified by mining 689 227 microbial genomes from NCBI GenBank using antiSMASH (Blin *et al.* 2019). After mapping the length-34 amino acid signature of these A-domains to RaptorX features, K-means clustering was conducted using euclidean distance and K=200 [decided via the elbow method (Bholowalia and Kumar 2014)]. Figure 3a shows a 2D embedding of all the A-domains using the t-SNE method. All A-domains belonging to the same cluster are given the same color. Figure 3b shows the labeled and unlabeled A-domains. Supplementary Fig. S4a shows that A-domains are not separable based on cultivability, and Supplementary Fig. S4b shows that A-domains are not separable based on their phylum.

Among the largest 50 clusters, 19 clusters do not contain A-domains with known labels. Several of these clusters are readily observable in Fig. 3. Clusters in the upper middle of Fig. 3a do not have labeled A-domains in Fig. 3b. These clusters with unlabeled A-domains likely represent novel amino acids.

### 3.5 Predicting substrate properties

In case of novel substrate specificities (e.g. novel amino acids), classification techniques are unable to provide information about the specificity. In these cases, instead of the identity of the suspects, their properties can be predicted. Table 2 shows the accuracy of various machine learning techniques in predicting different physiochemical properties of the substrate, including hydrophobicity, polarity, charge, aromaticity, presence of carboxyl and hydroxyl groups, and the number of atoms in the side chain. Our results show that the extra tree method achieves high accuracy in all these predictions. Currently, NRPSpredictor2 can only predict hydrophobicity. Table 3 compares the F1 scores of hydrophobicity classification for three different types of substrates: hydrophobic, hydrophilic aliphatic, and hydrophilic aromatic amino acids. We observed that almost all classifiers produced similarly accurate results for hydrophilic substrates, whereas NRPSPredictor2 is around 6% and 18% less accurate in case of hydrophobic aliphatic and aromatic substrates, respectively.

**Table 2.** Accuracy of different classifiers in predicting categorical amino acid attributes using one-hot encoding features.^a^

Accuracy	Polarity	Charge	Aromaticity	Carboxyl	Hydroxyl	Side chain
Logistic regression	0.86	0.88	0.85	0.92	0.92	0.84
k-Nearest neighbor	0.87	0.89	0.84	0.92	0.90	0.84
Multilayer perceptron	0.84	0.88	0.85	0.93	0.93	0.83
Ridge cross-validation	0.86	0.87	0.87	0.93	0.93	0.85
Ridge	0.85	0.87	0.84	0.91	0.9	0.82
Extra tree	0.86	0.89	0.84	0.93	0.93	0.85

a The attributes from left to right are substrate polarity, substrate charge, substrate aromaticity, whether substrate contains a carboxyl group, whether substrate contains a hydroxyl group, and whether the substrate side chain contains more than four atoms.

**Table 3.** F1 scores of different classifiers in predicting hydrophobic characteristics.^a^

Accuracy	Hydrophilic	Hydrophobic aliphatic	Hydrophobic aromatic
Logistic regression	0.68	0.86	0.78
k-Nearest neighbor	0.72	0.85	0.78
Multilayer perceptron	0.67	0.84	0.78
Ridge cross-validation	0.71	0.87	0.79
Ridge	0.69	0.85	0.77
Extra tree	0.70	0.87	0.79
NRPSPredictor2	0.69	0.8	0.60

a Classifiers are using one-hot encoding features.

## 4 Discussion

Currently, hundreds of thousands of NRP Biosynethetic Gene Clusters (BGCs) have been identified from microbial genomes. However, the molecular structure of the NRPs encoded by the majority of these BGCs have not yet been determined. During the past two decades, various machine learning approaches have been developed to predict the amino acids present in the molecular products of these BGCs based on the amino acid sequence of their A-domains. These methods use physiochemical properties of the amino acids in the binding pockets of A-domains to predict substrate specificity. However, it remains unclear whether these features improve the accuracy of classification. In this study, we show that these features alone do not provide any advantages in specificity prediction accuracy and simpler features that encode the identity of amino acids resulted in similar or better performance. Furthermore, for both amino acid physicochemical and amino acid identity features, NRPSPredictor2 is not the most performant model. This is because on the task of substrate specificity prediction, the extra trees and logistic regression model perform better than SVMs, the machine learning model that NRPSPredictor2 is based on.

We further observe that the accuracy of existing methods dropped for test A-domains that are further away (in terms of Hamming distance) from the training A-domains. The current evaluation metrics that measure the overall accuracy based on the training A-domains are unable to capture such deficiencies. Therefore, we presented a new evaluation metric to measure robustness of various machine learning techniques for the test data that is dissimilar to the training data. We showed random forest and extra trees have the highest robustness in comparison to the other methods, and further this robustness can be enhanced for test data points far away from the training data by integrating physiochemical and Alphafold2 features with amino acid identity features. NRPs are complex molecules synthesized by multiple enzymes in NRPSs. The intricate assembly process of NRPS makes it challenging to predict the final peptide product. However, by enhancing the accuracy of adenylation domain specificity prediction, we can determine which specific monomer each domain recruits. This enables us to generate hypothetical backbones and determine the final molecular structure more precisely. We believe that our work will serve as a valuable tool for researchers to gain a better understanding of the NRP synthesis mechanism, leading to the discovery of novel NRP molecules.

The unsupervised learning framework in the AdenPredictor toolkit identifies 19 clusters of A-domains that could potentially correspond to amino acids not previously implicated in NRPS assembly. These A-domains belong to cultivable bacteria, making them a gold mine for future discovery of NRPs with novel modes of action. The potential to discover novel mechanisms of action makes metabolites produced by these bacteria promising antibiotic candidates. AdenPredictor further predicts various properties of their substrates including hydrophobicity, polarity, charge, aromaticity, presence of carboxyl and hydroxyl groups, and the number of atoms in the side chain.

AdenPredictor’s extra trees machine learning model significantly improves the prediction accuracy of the specificity of adenylation domains in NRPs. Integration of AdenPredictor with mass spectrometry search techniques, e.g. NRPquest (Mohimani *et al.* 2014) and NRPminer (Behsaz *et al.* 2021) can accelerate the automated discovery of novel NRPs.

## Supplementary Material

btad235_Supplementary_Data

## Data Availability

The results present in this study are available from https://github.com/mmongiacmu/AdenPredictor. Data for supervised learning and unsupervised is available at https://github.com/mmongiacmu/AdenPredictor/tree/ma-in/supervised_preprocessing and https://github.com/mmongiacmu/AdenPredictor/tree/main/unsupervised_preprocessing, respectively.
