# Supplementary material for: AdenPredictor: accurate prediction of the adenylation domain specificity of nonribosomal peptide biosynthetic gene clusters in microbial genomes
Source: Bioinformatics. 2023 Jun 30;39(Suppl 1):i40–6. doi: 10.1093/bioinformatics/btad235 (PMC10311338; doi:10.1093/bioinformatics/btad235)
Supplement: btad235_Supplementary_Data [file btad235_supplementary_data.pdf]

Supporting Information: AdenPredictor: Accurate prediction of the adenylation domain specificity of nonribosomal peptide Biosynthetic Gene Clusters in Microbial Genomes

Mihir Mongia [1,2] Romel Baral [1,2] Abhinav Adduri[2]
Donghui Yan[2] Yudong Liu [2] Yuying Bian[2] Paul Kim[2]
Bahar Behsaz[2] Hosein Mohimani[2,3]

May 2022

Contents

S1 Machine Learning Classifier Parameters **1**

S2 Figures **3**

S3 Physiochemical Properties of Amino Acids **7**

S4 Test data splits by buckets **8**

S5 Extracting length-34 signature **8**

S6 Balance of Dataset Labels **9**

S1 Machine Learning Classifier Parameters

The parameter details for scikit-learn based classifiers are given as follows.

[1]These authors contributed equally to this work
[2]Computational Biology Department, School of Computer Science, Carnegie Mellon University, Pittsburgh, Pennsylvania
[3]Email: hoseinm@andrew.cmu.edu

- Logistic regression: random_state=0, max_iter=400, multi_class="multinomial", solver="newton-cg"
- Support vector machine: random_state=0, multi_class="crammer_singer", tol=1e-9, max_iter=2000
- K nearest neighbor: weights="distance"
- Multi layer perceptron: random_state=1, max_iter=400, early_stopping=False
- Random forest: max_depth=4, criterion="entropy"
- Decision tree: random_state=0, criterion="entropy"
- Bernoulli naive Bayes: default parameters
- Extra tree: n_estimators=100, random_state=0, criterion="gini"
- Gaussian naive Bayes: default parameters
- Label propagation: kernel="knn"
- Label spreading: kernel="knn"
- Linear discriminant analysis: default parameters
- Ridge cross validation: default parameters
- N centroid: default parameters
- Ridge: default parameters

S2 Figures

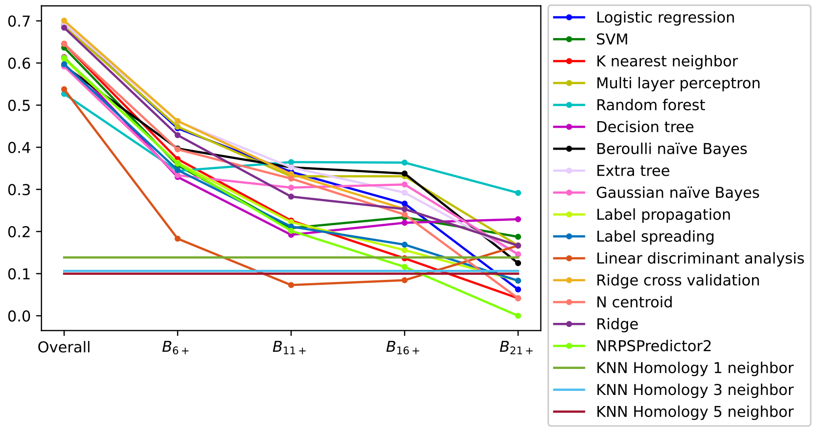

(a)

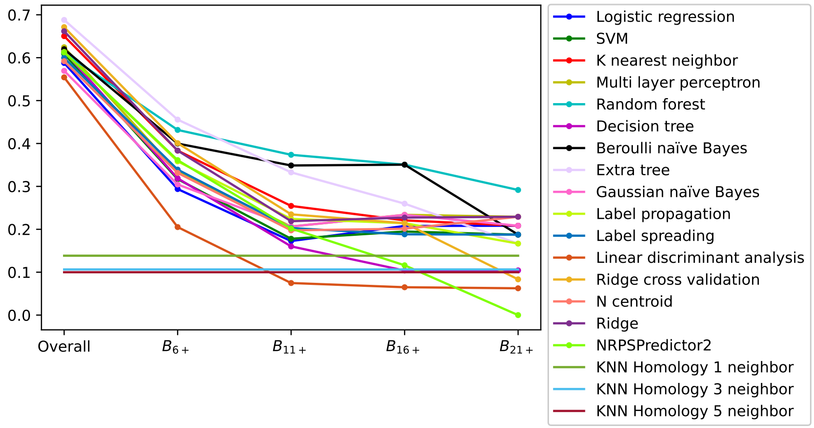

(b)

Figure S1: Accuracy of different classifiers using (a) one-hot encoding, and (b) physiochemical features. In KNN Homology 'x' neighbor classifier, label of each test data point is denoted by the label with maximum vote from x closest neighbors from training data points. Distance measure is edit distance computed with respect to full sequences of A-domains.

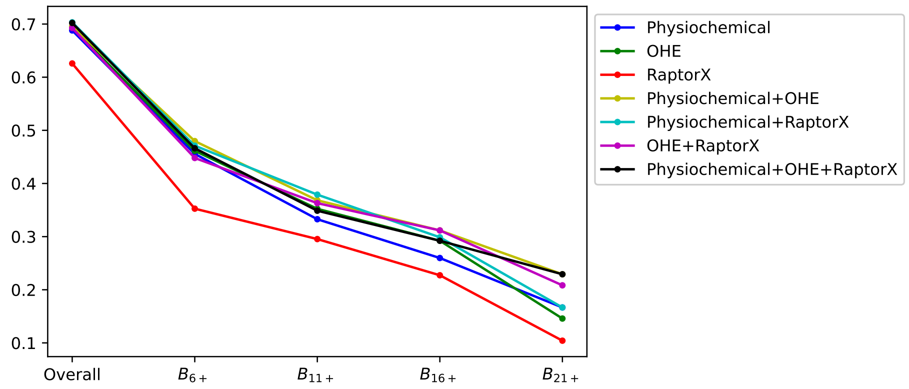

Figure S2: Comparison between different encoding techniques with extra tree classifier. OHE stands for one-hot encoding.

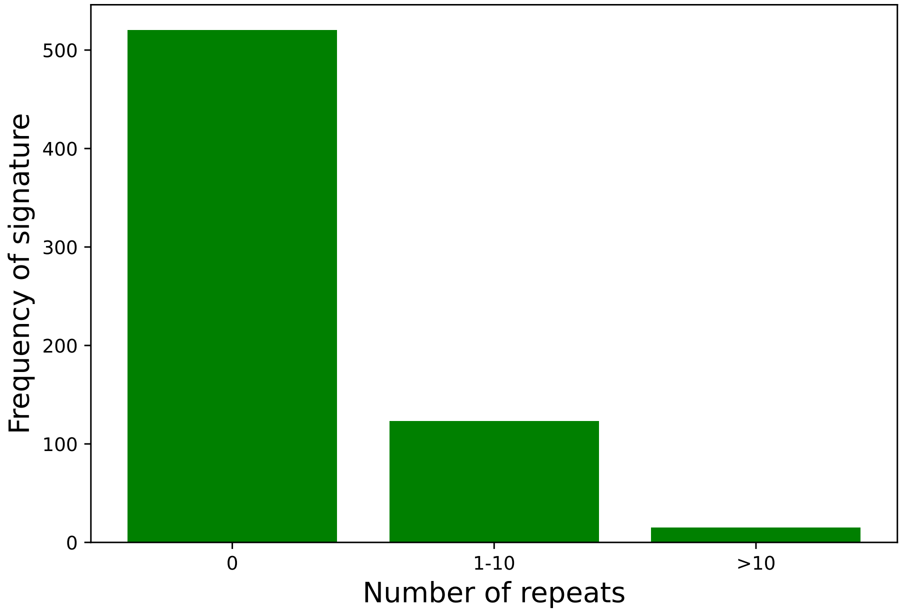

Figure S3: Frequency of observation vs number of repeats of various signatures

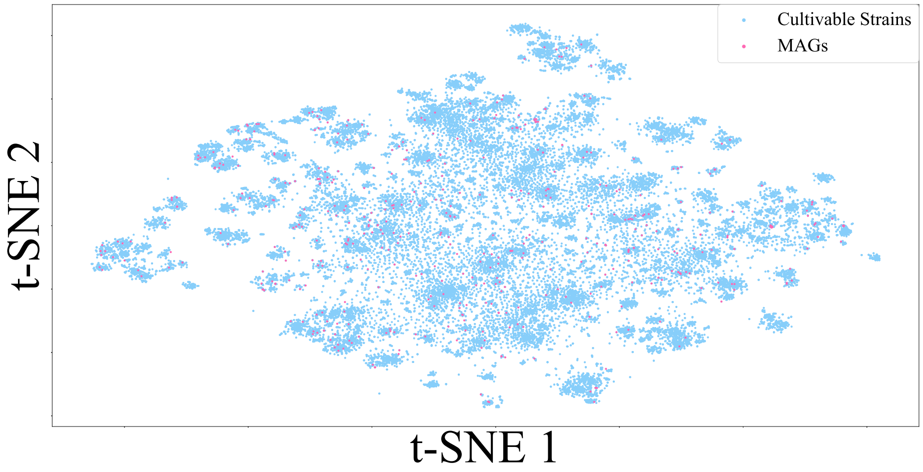

(a)

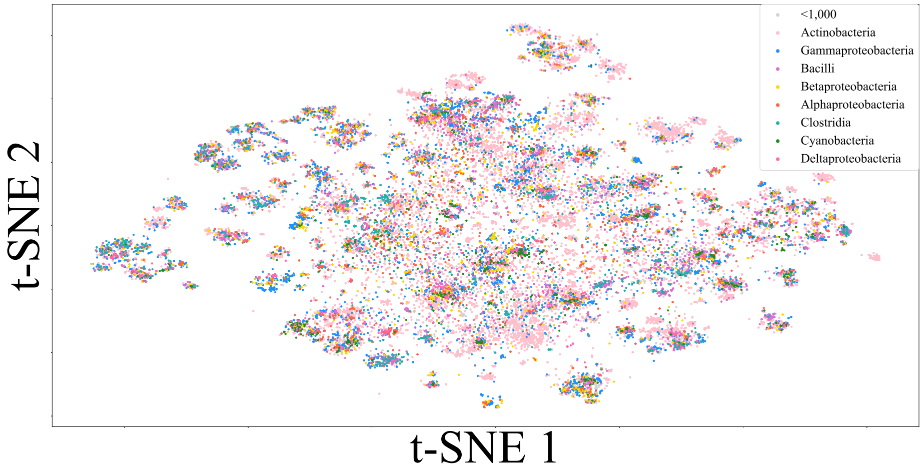

(b)

Figure S4: S4a shows that A-domains are not separable based on cultivability. S4b t-SNE visualization of all the clusters based on their phylum data. <1,000 refers to phyla with less than 1,000 samples in dataset. No separation based on phylum is observed. .

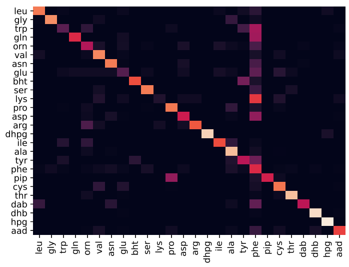

(a)

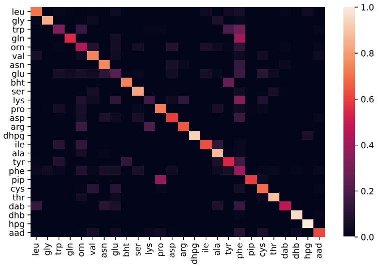

(b)

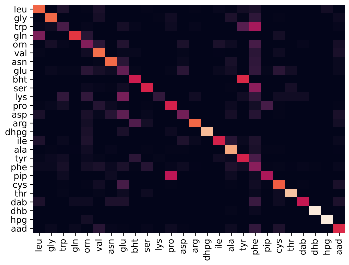

(c)

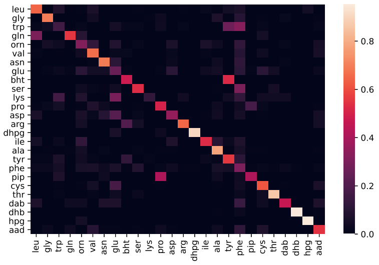

(d)

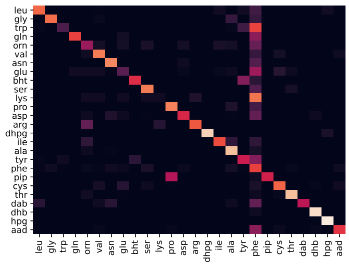

(e)

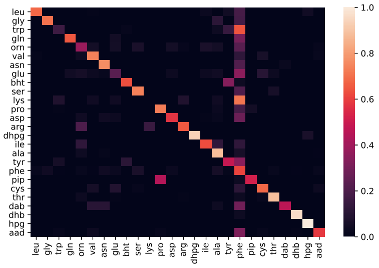

(f)

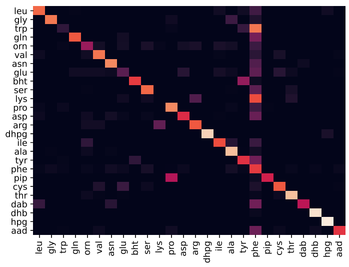

(g)

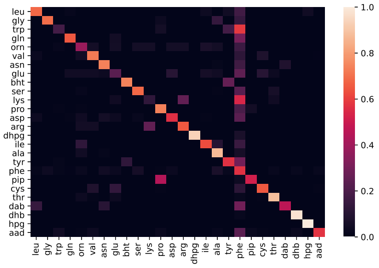

(h)

Figure S5: Confusion matrix for (a) logistic regression with OHE, (c) logistic regression with physiochemical encoding, (e) extra tree with OHE, (g) extra tree with physiochemical encoding. Figures (b,d,f,h) show confusion matrices after weight balancing in respective order.

S3 Physiochemical Properties of Amino Acids

aa	aa	rf1	rf2	rf3	rf4	rf5	rf6	rf7	hnmr1	hnmr2	hnmr3	vdw	mw	z1	z2	z3
A	Ala	60	24	29	9	52	23	37	4A3	3.77	3.3	13.7	89.1	0.07	-1.73	0.09
V	Val	74	42	43	22	68	43	72	3.94	3.6	3.03	34.1	117.2	-2.69	-2.53	-1.29
L	Leu	82	52	50	33	73	53	83	4.05	3.71	3.23	44.4	131.2	-4.19	-1.03	-0.98
I	ile	80	50	49	28	72	53	83	4.02	3.66	3.08	44.4	131.2	-4.44	-1.68	-1.03
P	Pro	52	20	50	11	57	31	68	4.37	4.11	3.51	30.7	115.1	-1.22	0.88	2.23
F	Phe	82	50	52	35	66	52	87	4.31	3.98	3.48	56.1	165.2	-4.92	1.3	0.45
W	Trp	86	54	54	40	-	51	88	4.36	4.05	3.56	75.1	204.2	-4.75	3.65	0.85
M	Met	77	45	50	29	63	49	79	4.2	3.84	3.3	45	149.2	-2.49	-0.27	-0.41
K	Lys	1	7	2	1	19	7	1	4.05	3.74	3.27	51.1	147	2.84	1.41	-3.14
R	Arg	1	9	6	1	19	7	1	4.06	3.76	3.2	64.9	175	2.88	2.52	-3.44
H	His	9	6	16	1	16	20	2	4.29	4	3.47	45.1	156	2.41	1.74	1.11
G	Gly	44	19	22	6	32	22	17	3.88	3.54	3.15	3.5	75.1	2.23	-5.36	0.3
S	Ser	44	19	15	6	36	23	13	4.18	3.87	3.31	18.3	105.1	1.96	-1.63	0.57
T	Thr	58	25	27	11	46	29	30	3.97	3.57	3.08	28.5	119.1	0.92	-2.09	-1.4
C	Cys	-	23	27	-	46	-	-	4.3	3.97	3	25	121.2	0.71	-0.97	4.13
Y	Tyr	81	50	47	32	68	51	80	4.3	3.9	3.4	61.6	181.2	-1.39	2.32	0.01
N	Asn	39	14	25	5	21	12	12	4.33	4	3.56	32.5	132.1	3.22	1.45	0.84
Q	Gln	53	16	37	7	32	20	23	4.1	3.76	3.24	42.7	146.1	2.18	0.53	-1.14
D	Asp	-	16	9	2	38	20	19	4.34	3.89	3.51	30	132.1	3.64	1.13	2.36
E	Glu	-	24	15	4	42	19	28	4.12	3.74	3.21	40.2	146.1	3.08	0.39	-0.07

Suppplementary Table 1: Physiochemical Properties used for NRPSPredictor2.

S4 Test data splits by buckets

Bucket Number	Percentage of Test Data
1	21.92982456
2	14.49275362
3	11.67048055
4	5.14874142
5	7.17009916
6	3.3180778
7	4.04271548
8	3.77574371
9	3.05110603
10	3.96643783
11	3.1655225
12	3.73760488
13	3.3180778
14	2.402746
15	2.93668955
16	1.90694127
17	0.49580473
18	0.87719298
19	0.1525553
20	0.61022121
21	0.49580473
22	0.80091533
23	0.26697178
24	0.26697178

Suppplementary Table 2: Percentage of test data belonging to different buckets across 20 instantiations of the test data. In each instantiation, 20 percent of data set is randomly assigned to test data and 80 percent to training data. Buckets are define in main text in section entitled Machine Learning Metrics and Generalization.

S5 Extracting length-34 signature

Each A-domain is first aligned to NRPS A-domain AMP-binding (PFAM ID PF00501.21) with HMMER3. Then 34 residues thought to be part of the A-domain binding pocket are selected. The below function specifies how these 34 residues are chosen. For further details on A-domain preprocessing refer to https://github.com/mmongiacmu/AdenPredictor/tree/main/supervised preprocessing.

```
def extract_sig(Id, top, bottom, idx_list):
    try:
        s1, p1 = extractCharacters(Id, top, bottom, idx_list, "KGVmveHrnvvnlvkwl", [12, 15, 16])
        s2, p2 = extractCharacters(Id, top, bottom, idx_list, "LqfssAysFDaSvweifgaLLnGgt", [3,8,9,10,11,12,13,14,17])
        s3, p3 = extractCharacters(Id, top, bottom, idx_list, "iTvlnltPsl", [4,5])
```

```
        s4, p4 = extractCharacters(Id, top, bottom, idx_list, "LrrvlvGGEaL", [4,5,6,7,8])
        s5, p5 = extractCharacters(Id, top, bottom, idx_list, "liNaYGPTEtTVcaTi", [1,2,3,4,5,6,7,8,9,10,11,12,13,14,15])

        return s1+s2+s3+s4+s5, p1+p2+p3+p4+p5
    except:
        return '', []
```

S6 Balance of Dataset Labels

Below is a list of tuples. In each tuple there is an amino acid substrate and the number of datapoints in the dataset that bind to the substrate.

'P': 22, 'F': 99, 'N': 22, 'Q': 10, 'Y': 22, 'V': 38, 'Orn': 5, 'L': 41, 'I': 15, 'C': 37, 'E': 32, 'K': 8, 'D': 28, 'T': 39, 'W': 16, 'G': 27, 'A': 618, 'S': 34, 'dhb': 266, 'aad': 70, 'orn': 11, 'pip': 9, 'bht': 9, 'hpg': 22, 'dhpg': 9, 'beta-ala': 5, 'dab': 12, 'R': 6, 'horn': 5, 'ORN': 1, 'hyv-d': 4, 'dht': 4
